# Neurovascular Pathophysiology and Emerging Biomarkers in Cerebral Malaria: An Integrative Perspective

**DOI:** 10.3390/neurolint17090149

**Published:** 2025-09-15

**Authors:** Damian Pikor, Mikołaj Hurła, Natalia Banaszek-Hurła, Alicja Drelichowska, Małgorzata Paul

**Affiliations:** 1 Department of Internal Medicine and Metabolic Disorders, University of Medical Sciences, Przybyszewskiego 49, 60-355 Poznan, Poland; 2 Department of Tropical and Parasitic Diseases, Central University Hospital, Przybyszewskiego 49, 61-701 Poznan, Poland; 3 Laboratory of Neurobiology, Department of Neurology, Poznan University of Medical Sciences, 60-355 Poznan, Poland; 4 The Student Scientific Society, Poznan University of Medical Sciences, Rokietnicka 5, 60-806 Poznan, Poland

**Keywords:** cerebral malaria, plasmodium falciparum, blood–brain barrier, neuroimaging

## Abstract

Cerebral malaria is a life-threatening neurological complication of *Plasmodium falciparum* infection and a leading cause of pediatric mortality in endemic regions of sub-Saharan Africa. It is defined clinically by coma accompanied by peripheral parasitemia, without alternative causes. Pathogenetically, cytoadherence of parasitized erythrocytes in the cerebral microvasculature, together with a widespread inflammatory response and endothelial activation, causes profound microvascular injury. This injury includes disruption of the blood–brain barrier and the development of multifactorial cerebral oedema (both vasogenic and cytotoxic), resulting in elevated intracranial pressure and often diffuse brain swelling as seen on imaging in fatal cases. Recent high-resolution MRI studies in pediatric cohorts from these endemic regions have identified characteristic neuroimaging findings such as basal ganglia infarcts, brainstem lesions, and corpus callosum abnormalities that strongly predict poor outcomes. Notably, circulating extracellular vesicles—released by parasitized erythrocytes and activated endothelial cells have emerged as potent mediators of microvascular inflammation. Extracellular vesicles contain parasite-derived antigens and host inflammatory signals, implicating them in disease mechanisms. These vesicles are under investigation as novel diagnostic and prognostic biomarkers for severe malaria. Importantly, survivors of cerebral malaria often endure persistent neurocognitive impairments, behavioral problems, and epilepsy, underscoring the need to prevent secondary neuronal injury during the acute phase to reduce long-term disability. Taken together, these insights highlight the interplay between cerebral microvascular pathology and neurological outcome in cerebral malaria. This review synthesizes recent advances in the pathophysiology of cerebral malaria and cutting-edge diagnostic modalities. It highlights novel therapeutic targets and neuroprotective strategies that may enable precision medicine approaches aimed at preventing lasting neurological disability in survivors.

## 1. Introduction

Cerebral malaria (CM) represents the most devastating neurological outcome of *Plasmodium falciparum* infection, tragically claiming hundreds of thousands of young lives annually across malaria-endemic regions [1,2]. Defined precisely, CM is diagnosed in a patient with confirmed *P. falciparum* parasitaemia who presents in an unrousable coma, with no other underlying cause [1]. While other *Plasmodium* species, such as *P. ovale* and *P. vivax*, can also induce malaria, *P. knowlesi* is considerably less common and its presence is largely restricted to Southeast Asia. Despite optimal antimalarial treatment and intensive care, childhood case-fatality rates for CM remain stubbornly high (15–25%) [1,2]. Furthermore, up to one-quarter of survivors are left grappling with chronic complications including persistent epilepsy, motor and sensory deficits, behavioral issues, and cognitive impairment [2,3]. The pathogenesis of CM fundamentally revolves around distinct neurovascular lesions. Mature *P. falciparum*-infected erythrocytes (iRBCs) express adhesive variant antigens, notably PfEMP1, which bind to endothelial receptors within the brain’s delicate microvasculature [4,5]. This critical sequestration leads to profound microvascular congestion, with nearly every cerebral microvessel becoming occluded by iRBCs or fibrin platelet thrombi [6]. The ensuing mechanical obstruction, compounded by localized inflammation and endothelial activation, precipitates severe microcirculatory impairment [4]. Anatomical pathology provides clear evidence of widespread vascular injury: the blood–brain barrier (BBB) is disrupted, perivascular hemorrhages appear, and both white and deep grey matter exhibit diffuse edema and axonal damage [6]. Advanced brain imaging techniques have unveiled characteristic lesion patterns in CM. High-resolution MRI in African children frequently shows involvement of the basal ganglia, presenting as focal infarcts or notable signal changes [7]. Children with visible retinal microvascular pathology invariably display MRI abnormalities, such as diffuse brain swelling and focal infarcts [8]. In adults with CM, diffusion-weighted MRI often demonstrates regions of restricted diffusion in the basal ganglia and corpus callosum, frequently accompanied by microhemorrhages on susceptibility sequences [7]. Novel biomarkers offer crucial insights into the unique biological mechanisms of cerebral malaria. Extracellular vesicles (EVs), shed by infected erythrocytes, activated platelets, and endothelium, carry parasite antigens and host inflammatory mediators [9]. Intriguingly, circulating EV levels correlate directly with malaria severity [9]. While angiopoietins have been investigated as potential indicators of CM, their role as reliable biomarkers still requires further validation compared to other promising candidates [4]. Experimental adjunctive therapies, such as anticytokine agents or treatments aimed at stabilizing the vasculature, have been explored, though their clinical success has been limited [4,10]. An integrative neurovascular perspective emphasizes the necessity of both parasite-directed and host-directed interventions, precisely tailored by biomarkers [4]. Finally, precision diagnostics, including retinal imaging or sophisticated molecular biomarker panels, are enhancing the specificity of CM diagnosis, ensuring that adjunctive therapies are accurately targeted to genuine malaria-induced brain injury [8,9].

## 2. Neurovascular Pathophysiology

### 2.1. Parasite and Host Immune Interactions in Cerebral Malaria

**Figure 1:**
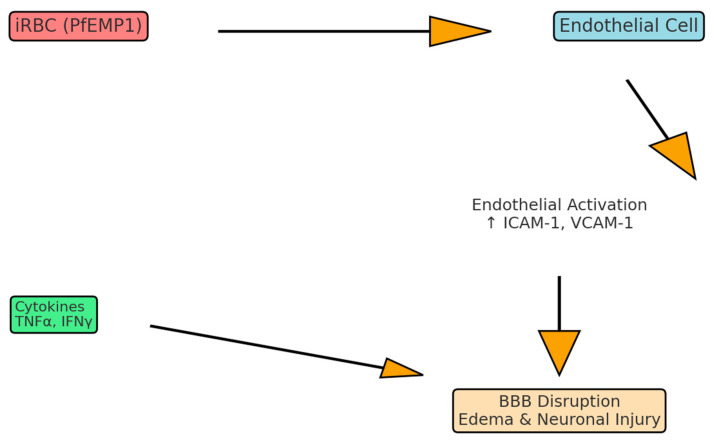
Pathophysiology of cerebral malaria. Schematic representation of the key pathogenic mechanisms in cerebral malaria. Infected erythrocytes expressing PfEMP1 adhere to endothelial receptors (ICAM-1, EPCR), leading to endothelial activation and upregulation of adhesion molecules. This promotes leukocyte recruitment, cytokine release (TNFα, IFNγ, CXCL10), and disruption of the blood–brain barrier. The combined effects result in cerebral edema, neuronal injury, and coma.

*Plasmodium falciparum*-infected erythrocytes (iRBCs), along with the associated inflammatory milieu, initiate a cascade of molecular events. These events drive endothelial activation, blood–brain barrier (BBB) disruption, and neurovascular injury [11,12]. Pro-inflammatory cytokines, notably TNFα, IFNγ, IL-1β, IL-6, and IL-8, are markedly elevated in severe CM cases. These cytokines upregulate endothelial adhesion molecules, thereby enhancing the sequestration of iRBCs and leukocytes within the cerebral microvasculature [12,13]. Chemokines such as CXCL10 (IP-10), which are induced by IFNγ and TNFα, further recruit activated monocytes and T cells to the brain endothelium. This amplifies local inflammation and contributes to vascular leakage [14,15]. These infiltrating immune cells release additional cytokines, proteases, and reactive oxygen species, collectively exacerbating perivascular edema and neuronal damage [12,16]. While anti-inflammatory mediators, including IL-10 and TGFβ, are upregulated in an attempt to curb inflammation, their effects are frequently insufficient amidst overwhelming pro-inflammatory stimuli [12,17]. In murine models, IFNγ receptor deficiency prevents cerebral manifestations by blocking endothelial antigen presentation to cytotoxic T cells, underscoring the critical role of adaptive immunity in CM pathology [18]. Indeed, recent histopathological analyses in pediatric CM have identified brain-infiltrating CD8+ T cells expressing perforin and granzyme B in proximity to sequestered parasites, directly implicating these lymphocytes in endothelial injury [19]. Cerebral malaria (CM) pathogenesis arises from a complex interplay between parasite-derived factors and a dysregulated host immune response at the neurovascular unit. In pediatric CM autopsies, CD8+ T cells expressing perforin and granzyme B have been observed adjacent to sequestered parasites, consistent with a role in endothelial injury [20] (Figure 1).

### 2.2. Endothelial Dysfunction and Blood–Brain Barrier Disruption

Circulating markers of endothelial activation, such as von Willebrand factor (vWF), soluble ICAM-1, VCAM-1, and endothelial microparticles, are significantly increased in severe malaria and possess considerable prognostic value [6,21]. Histopathological and imaging studies corroborate these molecular findings, revealing patchy BBB leakage, petechial hemorrhages, and vasogenic edema in fatal CM cases [19]. From a translational perspective, these markers have been explored as clinical biomarkers; for instance, high-plasma Ang-2 and soluble Tie2 (sTie2) levels independently predict cerebral microvascular pathology and mortality [6,13]. Although experimental strategies aimed at restoring endothelial stability, including modulation of the Ang/Tie2 axis and nitric oxide (NO) bioavailability, show promise in preclinical models, human trials, such as those involving inhaled NO, have thus far failed to demonstrate a survival benefit [22,23]. Endothelial dysfunction represents a central driver of neuropathology in CM. The synergistic effects of parasite sequestration and host-derived inflammatory mediators directly disrupt endothelial integrity and promote BBB breakdown [11,12]. A key feature of this perturbation involves the angiopoietin–Tie2 axis: Angiopoietin-1, which typically maintains endothelial quiescence, becomes depleted, while Angiopoietin-2, an antagonist of Ang-1, is elevated. This specific profile is strongly associated with disease severity, retinopathy, brain swelling, and fatal outcomes [6,13]. Ang-2 and related Tie2 axis markers associate with retinopathy and mortality, but effect sizes are modest in many cohorts (e.g., in Ugandan children, ANG-1 distinguished CM from UM with ~70% sensitivity and 75% specificity; AUCs for single markers often ≤0.71) [6,13,24].

### 2.3. Parasite Adhesion and Microvascular Sequestration

At the parasite–host interface, *P. falciparum* expresses variable PfEMP1 adhesins that bind to cerebral endothelial receptors, notably the endothelial protein C receptor (EPCR) and intercellular adhesion molecule-1 (ICAM-1) [24,25]. Specific PfEMP1 variants that engage both EPCR and ICAM-1 drive localized endothelial activation, inflammation, and coagulation disturbances by promoting EPCR shedding and impairing the protective protein C pathway [25,26]. These dual-binding parasites are highly correlated with pediatric CM cases presenting with severe brain swelling and a poor prognosis [24,25]. Additionally, the rosetting of iRBCs with uninfected erythrocytes contributes to microvascular occlusion and hypoperfusion [25]. Such extensive sequestration and aggregation exacerbate local hypoxia and metabolic stress, further fueling neuroinflammation and endothelial injury [25,27].

### 2.4. Cytokine Milieu, Chemokines, and Immune Dysregulation

The cytokine and chemokine milieu in CM is undeniably complex, involving numerous mediators acting in concert. IL-6, IL-8, MCP-1, and other pro-inflammatory factors are consistently elevated in severe cases, likely contributing to leukocyte recruitment and endothelial activation [12,13,15]. CXCL10 (IP-10), a chemokine induced by IFNγ, plays a key role in attracting T cells and monocytes to sites of inflammation; elevated IP-10 levels have been consistently linked with CM severity and unfavorable outcomes [14,28]. Despite these insights, therapeutic efforts targeting single cytokines have largely been unsuccessful in clinical settings, a consequence of the intricate balance between protective and pathogenic immune responses [29]. While experimental blockade of lymphotoxin-α or its receptor can protect against murine CM, such interventions carry the risk of impairing parasite control in humans [18,29].

### 2.5. Translational Biomarkers and Adjunctive Therapy Development

The clinical translation of these mechanistic insights has primarily focused on identifying reliable biomarkers and developing adjunctive therapies. Plasma markers such as Ang-2, sICAM-1, sVCAM-1, vWF, TNFα, IFNγ, and CXCL10 offer significant potential for early risk stratification and prognosis in CM [6,13,14,28]. Developing molecular panels that combine these markers may further enhance diagnostic specificity and guide targeted interventions [6,14]. Adjunctive therapies aimed at stabilizing the vasculature (e.g., Ang-1 mimetics, NO donors) or modulating immune responses (e.g., CXCR3 blockade, anti-platelet strategies) have shown promise in preclinical studies. However, their efficacy in human trials remains unproven [22,23,29]. The notable failure of numerous adjunctive agents, including corticosteroids, anti-cytokine antibodies, and aspirin, underscores the inherent challenge of effectively targeting complex and often redundant pathogenic pathways in CM [29].

## 3. Biomarkers

### 3.1. The Angiopoietin-Tie2 Regulatory System

Angiopoietin-2 (Ang-2) is higher and Angiopoietin-1 (Ang-1) is lower in cerebral malaria (CM) than in uncomplicated malaria, according to several studies. This results in a high Ang-2/Ang-1 ratio that effectively separates CM from less severe infections [6]. The Ang-2/Ang-1 ratio (or low Ang-1 alone) effectively distinguished CM from uncomplicated malaria in both Thai adults and Ugandan children, according to ROC analysis [30]. For instance, Ang-1 has 100% sensitivity and specificity (when <15 ng/mL) to detect CM in Thai adults, and 70%/75% sensitivity and specificity in Ugandan children, according to one study [31]. Significantly, in African children with CM, elevated admission Ang-2 levels predict death and the degree of retinopathy [13,32]. Ang-2 and Ang-1 levels were considerably greater and lower, respectively, in children with retinopathy (an indication of real CM) in Malawian pediatric CM [33], and high Ang-2 (cut-point ~3.9 ng/mL) was an independent predictor of death (adjusted OR = 7.9, *p* < 0.001) [34]. On the other hand, Ang-1 levels consistently increase as patients recover clinically, indicating that endothelial support has been restored. This highlights Ang-1’s function in preserving the endothelium barrier throughout recovery [35]. Mechanistically, endothelial cells’ Weibel–Palade structures store Ang-2, which is released upon activation and makes the endothelium more sensitive to inflammatory stimuli [36]. By encouraging inflammation and endothelial destabilization, Ang-2 generates a harmful positive feedback loop in CM. As a result, one of the endothelium biomarker pathways in severe malaria that has been studied the most is the angiopoietin–Tie2 axis [37].

### 3.2. Cell-Adhesion Molecules and Von Willebrand Factor

Elevated soluble adhesion molecules in CM also indicate endothelial activity. In certain cohorts, circulating sICAM-1 and sVCAM-1 levels significantly increase in CM, correlate with the depth of coma, and differentiate even asymptomatic parasitemia from uninfected controls. One characteristic of CM disease is the sequestration of infected erythrocytes in brain microvessels, which is made easier by these adhesion molecules. The hemostatic glycoprotein von Willebrand factor (vWF) and its propeptide (VWFpp) are also markedly increased in CM, indicating endothelial damage and abrupt Weibel–Palade body release. The vWF-cleaving protease, ADAMTS13, is reduced in CM patients and inversely correlates with endothelial activity, indicating that excess vWF directly leads to cerebral microangiopathy. Given that mice without VWF had a higher survival rate in cases of cerebral malaria, experimental models suggest that vWF plays a pathogenic function. High levels of sICAM-1, sVCAM-1, vWF, and VWFpp are well-validated biomarkers of endothelial dysfunction in CM, contributing to microvascular obstruction and blood–brain barrier breakdown [38].

### 3.3. Chemokine Profiles and Immune Activation

In deadly CM, pro-inflammatory chemokines are regularly elevated. Specifically, one of the most consistently increased biomarkers in CM is the interferon-inducible chemokine CXCL10/IP-10. CXCL10 (IP-10) levels are higher in fatal CM in some cohorts, but reported effect sizes vary: in Ghana, associations with death were significant without reported AUC, whereas in an Indian cohort (*n* ≈ 6 HC/26 MM/26 CM survivors/12 CM non-survivors), ROC AUCs approached 1.0 but sample sizes were small, warranting caution [39,40]. CXCL10 and platelet factor-4 (CXCL4) measurements together were found to perfectly differentiate between fatal CM and survivors (AUC = 1.0) in one noteworthy study [28]. CXCL10 levels are consistently greater in malaria patients than in healthy controls, according to a recent systematic review (26 research, 1933 participants), and in the majority of investigations, higher CXCL10 levels were associated with more severe disease [41]. In terms of biology, CXCL10 is not merely a bystander; in vitro, recombinant CXCL10 causes dose-dependent apoptosis in neural and microvascular endothelial cells in the human brain, directly linking it to neurotoxicity and breakdown of the blood–brain barrier. In animal models, type I interferon (IFNβ) is produced when endothelial sensing of parasite-derived heme via STING1 occurs. This leads to the release of CXCL10, the recruitment of leukocytes, and fatal inflammation of the brain [42]. Therefore, CXCL10 is a mediator of CM pathogenesis (a marker and creator of neuropathology) as well as a potent prognostic biomarker.

### 3.4. STING1-IFNβ-CXCL10 Axis and Innate Immune Sensing

**Figure 2:**
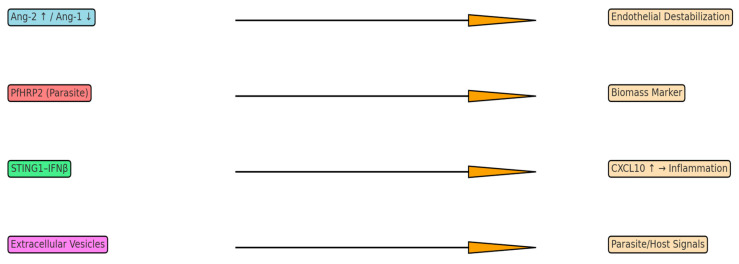
Biomarker pathways in cerebral malaria. Overview of major host–parasite biomarker axes implicated in cerebral malaria. The angiopoietin–Tie2 axis (Ang-2↑/Ang-1↓) destabilizes the endothelium, while the STING1–IFNβ–CXCL10 pathway drives neuroinflammation and blood–brain barrier breakdown. PfHRP2 reflects total parasite biomass and contributes directly to endothelial dysfunction. Circulating extracellular vesicles (EVs) carry parasite antigens and host inflammatory mediators, serving as emerging diagnostic and prognostic tools.

Recent mechanistic studies in murine models have revealed that brain endothelial cells directly sense parasite products via the STING1 pathway. Heme from sequestered *Plasmodium* parasites acts as an alarmin that activates STING1 in cerebral endothelial cells, leading to IFNβ transcription and subsequent CXCL10 induction [43]. This STING1/IFNβ/CXCL10 cascade drives leukocyte infiltration and blood–brain barrier breakdown, determining CM lethality. Crucially, mice with brain endothelium-specific STING1 deletion are fully protected from experimental CM despite similar parasite loads [44]. This highlights endothelial innate immunity as a key pathological sensor and potential therapeutic target. In parallel, STING activation in T cells modulates anti-inflammatory responses. A recent in vivo study demonstrated that during blood-stage *P. falciparum* infection, STING in CD4+ T cells induces autocrine type I IFN (IFNβ) which drives development of IL-10^ + IFNγ^ + type 1 regulatory (Tr1) T cells [45,46]. Thus, STING signaling has pleiotropic roles in malaria: in endothelium, it amplifies harmful inflammation (CXCL10-driven), while in T cells, it promotes immunoregulatory Tr1 responses. These findings underscore the complexity of innate immune sensing in CM pathogenesis and may inform novel interventions (Figure 2).

### 3.5. Parasite-Derived Biomass Indicators

A parasite-derived antigen called plasma PfHRP2 measures the overall body parasite biomass, including sequestered parasites. PfHRP2 levels increase with the overall infection load, in contrast to peripheral parasitemia, which understates sequestered burden. Despite equal parasite levels, the mean admission PfHRP2 in fatal cases of the historic AQUAMAT trial (3826 African children with severe malaria) was around 1611 ng/mL, while in survivors, it was approximately 1046 ng/mL [47]. This demonstrates the predictive significance of PfHRP2 outside of microscopy. A U-shaped mortality connection is shown with PfHRP2. True severe malaria in AQUAMAT is identified by concentrations above ~174 ng/mL; death rose significantly with PfHRP2 > 174 ng/mL (adjusted chances increased ~20% per log increase) [48]. Many youngsters probably suffered from non-malarial illnesses with incidental parasitemia below this threshold. PfHRP2 can thereby increase the accuracy of severe malaria diagnosis and stratify risk [49]. PfHRP2 is more than just a biomarker; it could be harmful in and of itself. PfHRP2 circulates as a heme-laden multimer, forming 14-mer nanoparticles with up to 700 heme groups per particle, according to recent research [50]. Brain endothelial cells internalize these PfHRP2–heme complexes (via caveolin-mediated endocytosis), and the released heme causes the endothelial barrier to break down by activating the NLRP3 inflammasome and releasing IL-1β [51]. Through inflammasome-mediated junctional rearrangements, recombinant PfHRP2 (at pathologic doses) damages human brain endothelial monolayers in vitro [52]. PfHRP2 nanoparticle-infused mice exhibit accelerated lethal malaria pathogenesis and blood–brain barrier leakage in vivo. Therefore, PfHRP2 serves as a virulence factor that causes endothelium inflammation as well as a quantitative indicator of parasite load [53,54].

### 3.6. Retinopathy Associated Patterns and Diagnostic Specificity

One particular indication of CM pathology is malarial retinopathy, which manifests as retinal hemorrhages and whitening. Plasma PfHRP2 concentrations are higher in retinopathy-positive CM and reflect total parasite biomass, including sequestered parasites; peripheral parasitemia underestimates hidden biomass, so higher PfHRP2 aligns with greater disease severity [6,47]. PfHRP2 can therefore be used in conjunction with other markers to enhance diagnosis. A panel of platelet count < 150,000/μL and PfHRP2 > 1000 ng/mL demonstrated 74% sensitivity and 93% specificity for “true” severe malaria, according to a recent latent class analysis (four trials, 2649 patients) [55]. According to this concept, almost one-third of African children who receive a clinical diagnosis of severe malaria actually suffer from another ailment. These findings suggest that in environments with limited resources, CM diagnosis can be improved by combining parasite-derived biomarkers (PfHRP2) with host indicators (platelets) [55] (Table 1).

## 4. Integrative Perspective on Pathophysiology and Biomarkers

### 4.1. Conceptualizing Systematic Approaches to Cerebral Malaria

Current research in cerebral malaria (CM) is emphasizing a crucial shift: moving past simplistic linear models to embrace systems-based analyses [56]. This fundamental change means that instead of scrutinizing one protein at a time, researchers are now examining the coordinated behavior of entire molecular networks. For example, genome-wide approaches like weighted gene co-expression network analysis (WGCNA) have already revealed host gene modules intricately linked to CM outcomes [56]. In practice, applying machine learning and network inference to multi-omic data has helped identify critical gene clusters and pathways relevant to the disease (e.g., MBP, SAMSN1, PSMF1, SLC39A8) that might have been overlooked by conventional methods [56]. Such approaches strongly suggest that survival and pathogenesis are governed by complex host–parasite interactions and metabolic pathways, which would remain hidden if only single biomarkers were examined in isolation. Modern algorithms are also adept at uncovering subtle patterns in routine data. For instance, deep convolutional neural networks (CNNs) can detect malaria parasites on blood films with remarkable accuracy, approaching 99% [57]. Similarly, computer-vision models (Mask R-CNN) can count infected cells 15 times faster than manual microscopy without any loss of accuracy [58]. These compelling results imply that artificial intelligence methods can reveal previously hidden complexity within clinical parameters and significantly improve severity classification beyond the capabilities of traditional analysis.

### 4.2. Extracellular Vesicles as a Window into Brain Pathophysiology

Extracellular vesicles (EVs) mediate bidirectional cell-to-cell communication during falciparum malaria. EVs released from infected erythrocytes carry parasite proteins, lipids, and nucleic acids that are taken up by other parasites and host cells, modulating parasite development and host responses. In vitro exosome-like vesicles transfer genetic material between parasites and promote gametocytogenesis, providing a mechanism for population-level coordination [59]. Likewise, microvesicles from infected erythrocytes are internalized by recipient parasites and stimulate transmission-stage differentiation, and are also taken up by innate immune cells, triggering cytokine production [60]. EV cargo and release vary across the intraerythrocytic cycle and between strains/isolates, with stage- and strain-related differences in protein and RNA content that may influence virulence and biomarker performance [6,40]. Proteomic studies indicate that EV composition differs between early (ring) and late (trophozoite/schizont) stages and across clinical isolates, supporting the concept that EV signatures will reflect disease stage and host–parasite heterogeneity [40]. Recent studies have shown that circulating extracellular vesicles (EVs) carry tissue-specific RNA signatures, providing a novel, non-invasive glimpse into brain pathology. In patients with cerebral malaria, approximately 32.2% of plasma EV-RNA originates from solid organs, and strikingly, 37.7% of this solid-tissue fraction is derived directly from the brain [61]. This means that sequencing EV-RNA in blood can offer real-time insights into the transcriptional changes occurring within the brain. Deconvolution of EV transcriptomes from affected patients clearly demonstrates a progression of pathology: transcripts from neuronal genes progressively decline, while those from glial cells, endothelial cells, and immune cells increase as the disease worsens [62]. These patterns strongly correlate with clinical severity; for example, the “pseudotime” ordering of EV profiles aligns with key severity indicators such as metabolic acidosis and coma depth [63]. Moreover, the composition of EVs in late-stage disease points to specific pathogenic processes: gene sets related to platelet activation, TNF and VEGF signaling, neurotrophin signaling, and glutamatergic neurotransmission are enriched in EVs from patients with advanced cerebral malaria [64]. These findings suggest that EVs not only report ongoing brain injury but may also actively participate in the intercellular communication driving the pathology.

### 4.3. Revolution in Molecular Diagnostics

Malaria diagnostics are undergoing a rapid transformation, evolving from traditional microscopy-based tests to precise molecular assays. Contemporary approaches now leverage host gene expression signatures and biomarker panels to accurately classify disease severity [31]. For example, transcriptomic profiling of brain endothelium exposed to Plasmodium-infected erythrocytes identified specific markers: the metalloprotease ADAMTS18 emerged as the top discriminator between uncomplicated and severe malaria, and two others (angiopoietin-like 4 and Inhibin-βE) could distinguish cerebral malaria cases among severe infections [31]. Importantly, combining these markers into a panel significantly improved diagnostic performance [65]. Similarly, deep learning applied to blood smear images can detect infection and classify Plasmodium species with exceptionally high accuracy (up to ≈99% in recent studies [57]). Innovative image-based AI models like Mask R-CNN not only automate parasite detection but also generate quantitative segmentation masks for visualization, producing diagnostic reports 15-fold faster than manual counting [58]. These groundbreaking advances demonstrate that integrating multiplex molecular data and machine learning can create rapid, high-accuracy diagnostics far surpassing traditional approaches.

### 4.4. Novel Pathophysiological Mechanisms

Recent discoveries are fundamentally reshaping our understanding of cerebral malaria mechanisms and, in turn, revealing promising new therapeutic targets. A key finding is that the EphA2 receptor tyrosine kinase on brain endothelium mediates blood–brain barrier (BBB) breakdown in malaria. In mouse models, EphA2 is upregulated by inflammatory signals, and its presence is essential for junctional disassembly and CD8+ T-cell infiltration into the brain [4]. Crucially, blocking EphA2 (for instance, with existing anticancer drugs or engineered inhibitors) effectively protected mice from BBB disruption [4]. This breakthrough identifies EphA2 as a critical mediator of neurovascular injury and a compelling potential therapeutic target in cerebral malaria. Another significant breakthrough involves the elucidation of the IL-33–NLRP3 inflammasome axis in neuroprotection. In experimental cerebral malaria, administering recombinant IL-33 alongside standard antimalarial drugs markedly improved survival and reduced brain pathology [66]. Mechanistically, IL-33 suppressed the activation of the NLRP3 inflammasome and reduced pro-inflammatory IL-1β release from microglia and monocytes [66]. Similar benefits were observed when a specific NLRP3 inhibitor (MCC950) was used instead of IL-33, demonstrating that targeting this inflammasome pathway can restore the efficacy of antimalarial therapy and minimize neurological injury [67]. These findings suggest entirely novel therapeutic strategies: for example, adjunctive IL-33 or inflammasome inhibitors could prove effective even in advanced disease stages.

### 4.5. Clinical Translation Challenges

Despite these profound molecular insights, translating discoveries into practical clinical tools remains a significant challenge. Cerebral malaria manifests differently in young African children compared to adults or older children in Asia, and endemic settings vary considerably in transmission intensity and immunity levels [68]. This inherent heterogeneity means that candidate biomarkers and treatments absolutely must be rigorously validated across diverse age groups and geographical regions before widespread implementation. Additionally, resource constraints in malaria-endemic areas necessitate that any new diagnostic or therapeutic solution be both affordable and field-friendly. For instance, even simple host markers like elevated C-reactive protein and thrombocytopenia have shown surprisingly robust correlations with severe malaria in both immune and non-immune populations [69], making them useful starting points for point-of-care tests. However, these markers lack specificity for cerebral involvement, so more sophisticated yet practical assays must be developed. In essence, bridging the gap from laboratory bench to patient bedside will demand extensive clinical evaluation of biomarker panels and meticulous consideration of implementation strategies in low-resource settings.

### 4.6. Multi-Omic Data Integration

The future of understanding and combating cerebral malaria lies in integrating diverse omic datasets into unified predictive models. Single-cell and spatial transcriptomics are beginning to meticulously detail how specific brain cell types respond to malaria, offering unprecedented resolution on the localized processes of injury and repair. Simultaneously, machine learning methods continue to advance. For instance, combining WGCNA with regression approaches has already identified novel host gene signatures in cerebral malaria [56]. A recent study applied network analysis and differential expression to whole-blood profiles, highlighting nine key host genes (including MBP, SAMSN1, PSMF1, SLC39A8) strongly linked to cerebral malaria [56]. Moving forward, researchers aim to merge transcriptomic, proteomic, metabolomic, host genetic, and parasite genomic data. Large-scale analyses will be essential to truly understand how human and parasite genetic variants influence biomarker levels and disease susceptibility [70]. This comprehensive, integrative, personalized approach is expected to yield more precise biomarker panels and inform tailored prevention and treatment strategies for different at-risk populations.

## 5. Clinical Implications and Future Research Directions

### 5.1. Preventive Strategies and Current Vaccines

Integrated prevention remains foundational for reducing CM risk, including insecticide-treated nets, indoor residual spraying, prompt diagnosis and treatment, and seasonal malaria chemoprevention in eligible settings. Two WHO-recommended vaccines for Plasmodium falciparum—RTS, S/AS01 (recommended in 2021) and R21/Matrix-M (recommended in 2023)—are being rolled out in African countries as part of routine immunization programs [71,72]. These vaccines reduce clinical malaria episodes and severe disease in children and may, indirectly, lower the incidence of CM when combined with vector control and effective case management. Ongoing surveillance and implementation research are essential to quantify their population-level impact on CM and neurocognitive outcomes.

### 5.2. A New Era of Precision Medicine in Cerebral Malaria

While current antimalarial drugs effectively eradicate parasites, they do not directly address the host’s complex pathological responses within the brain [73,74,75]. Therefore, a new era of precision medicine in cerebral malaria demands the development of therapies guided by specific biological markers of disease mechanisms. The angiopoietin–Tie-2 pathway perfectly illustrates this paradigm. Patients with cerebral malaria frequently exhibit markedly high Angiopoietin-2–Angiopoietin-1 ratios, reflecting profound endothelial activation. This ratio not only correlates strongly with fatal outcomes [34] but also identifies individuals who might particularly benefit from treatments designed to stabilize the endothelium. In preclinical models, supplementing Angiopoietin-1, when added to standard antimalarials, effectively preserved the blood–brain barrier and significantly improved survival [34]. Similarly, plasma levels of chemokines can guide therapeutic decisions. In one study, elevated CXCL10 (in conjunction with CXCL4) perfectly discriminated fatal CM cases (achieving an area under the ROC curve of 1.0) [39]. In clinical practice, a patient presenting with rising CXCL10 levels could be a strong candidate for aggressive anti-inflammatory intervention. Another emerging example is quantitative parasite antigen testing: plasma PfHRP2 concentration has been validated as a reliable proxy for total parasite biomass, and a threshold around 174 ng/mL has been proposed to distinguish true severe malaria from incidental parasitemia [48]. In principle, these molecular criteria could refine treatment decisions (for instance, by identifying patients who require more intensive care), though deploying such precision diagnostics in field settings will necessitate overcoming significant logistical and cost barriers.

### 5.3. Diagnostic Revolution and Point-of-Care Applications

Technological advancements are fundamentally transforming the diagnostic landscape for cerebral malaria. Standard tests often cannot reliably detect ongoing brain dysfunction, highlighting an urgent need for rapid point-of-care (POC) assays. Here again, extracellular vesicles (EVs) show considerable promise: they carry parasite proteins and host inflammation markers that accurately reflect disease status [64]. For example, EVs derived from Plasmodium-infected erythrocytes or activated endothelium could potentially be used to detect or stratify infection. More immediately practical are combined marker panels. A large-scale study involving 2649 patients across Africa and Asia demonstrated that using just two simple measurements—a platelet count of ≤150,000/µL plus a plasma PfHRP2 level of ≥1000 ng/mL—could identify true severe malaria with high accuracy (approximately 74% sensitivity and 93% specificity) [55]. This multi-parameter rule significantly outperformed either marker used alone and would have flagged one-third of presumed severe malaria cases as likely attributable to other causes. Such combined host–parasite diagnostics could substantially improve case management and optimize resource allocation in endemic areas. Moving forward, the ideal POC devices will seamlessly integrate multiple indicators (such as parasite antigens and inflammatory proteins) into rapid, low-cost assays.

### 5.4. Therapeutic Target Identification and Drug Development

Immune targets, particularly single cytokines, have proven ineffective or even harmful when broadly administered. Instead, therapies are now being developed with the aim of stabilizing the vasculature and mitigating specific pathogenic pathways. Endothelial stabilization stands as a leading strategy: studies indicate that enhancing Tie-2 signaling (for example, with Angiopoietin-1 mimetics or nanoparticles) can effectively protect the blood–brain barrier [34]. Inhaled nitric oxide was also trialed for its vasodilatory and anti-inflammatory effects, but thus far, it has not demonstrated a clear survival benefit in clinical trials. Multiple studies implicate the MMP pathway: serum TIMP-1 and MMP-8 are elevated in falciparum malaria and TIMP-1 correlates with severity, and in pediatric CM retinal MMP-8 locally co-localizes with fibrinogen leak at sites of sequestration, consistent with endothelial barrier disruption [76,77]. Targeting this pathway, perhaps by modulating the MMP/TIMP balance, is an emerging concept. Parasite–host interaction targets are also highly promising: as previously mentioned, blockade of EphA2 has shown efficacy in preventing BBB breakdown in animal models [4]. Drugs or biologics that inhibit EphA2 or similar endothelial receptors could potentially become crucial adjunctive therapies to preserve neurological function. The limited success of many adjunctive therapies to date underscores the critical need for interventions grounded in detailed mechanistic understanding (Table 2).

### 5.5. Immune Modulation and Neuroprotective Strategies

The immune response in cerebral malaria is a double-edged sword, and new insights are driving refined modulation strategies. CD8+ T cells contribute to microvascular injury in experimental models and are present near sequestered parasites in pediatric CM autopsies, but their causal role in human CM requires further study [20]. These cells rely on EphA2-mediated signals to enter the brain [78], making blocking their recruitment a viable approach (again highlighting EphA2 as a key target). Simultaneously, inflammasome pathways offer a promising point of intervention. As noted, blocking the NLRP3 inflammasome with IL-33 or specific inhibitors reduced brain inflammation and significantly improved outcomes in mice [66]. Crucially, these interventions also prevented chronic neurological deficits in survivors. A strikingly different approach has been the application of cell therapy. In mice that recovered from experimental cerebral malaria, a single dose of mesenchymal stem/stromal cells (MSCs) as an adjuvant to antimalarials conferred systemic benefits: it protected against vascular damage, restored brain neurotrophic factors, and ameliorated depression-like behaviors [79]. While MSC treatment did not directly reduce brain inflammation or parasite load, it notably improved survival and multi-organ function (especially in the lung and kidney). These findings suggest that augmenting endogenous repair mechanisms (for example, via MSCs or other neuroprotective agents) could effectively complement parasiticidal treatment and help prevent long-term sequelae.

### 5.6. Long-Term Outcomes and Rehabilitation Strategies

The increasing recognition of persistent deficits after malaria has led to a greater emphasis on long-term care. Approximately 25–50% of children who survive cerebral malaria experience lasting neurological or cognitive impairment [80]. Risk factors for these enduring issues include severe acute features such as prolonged coma, multiple seizures, hypoglycemia, and signs of raised intracranial pressure. These factors likely reflect the initial severity of brain injury. Furthermore, evidence suggests that the vascular and inflammatory abnormalities often do not fully normalize even after parasite clearance. For example, patients who maintain high plasma Angiopoietin-2 levels through convalescence exhibit worse recovery trajectories (persistent endothelial activation increases the risk of both fatal and non-fatal complications) [81]. Animal studies also confirm long-term effects: mice cured of experimental cerebral malaria still display cognitive deficits a month later, and such neurological dysfunction can persist for years in human survivors. Given this reality, adjunctive therapies should aim not only to save lives but also to actively protect the brain. Early screening for cognitive deficits and the implementation of rehabilitation therapies (including educational support, cognitive training, and physical therapy) may become an integral part of comprehensive cerebral malaria care.

### 5.7. Future Research Priorities and Technological Innovations

Future progress in combating cerebral malaria will fundamentally rely on the seamless integration of cutting-edge technology with translational science. High-resolution atlases of brain responses (including single-cell and spatial transcriptomics) are precisely mapping how different cell types react to malaria over time. Artificial intelligence and bioinformatics will continue to be indispensable for mining these vast datasets. For example, recent work combining WGCNA with gene expression analysis identified several novel host genes (MBP, SAMSN1, PSMF1, SLC39A8) as promising candidates for therapeutic targeting [56]. As omic datasets continue to accumulate, machine learning will be crucial for discovering optimal biomarker signatures. Crucially, all promising markers and therapies must undergo rigorous validation across the diverse spectrum of malaria settings. Genetic variation in human populations (such as the sickle cell trait or differences in immune genes) and in parasite strains can alter disease presentation and biomarker levels [82]. Therefore, large multi-center studies are essential to confirm that diagnostic thresholds and treatment strategies are effective in African children, Asian adults, and other affected groups alike. The integration of host and parasite genomics, coupled with environmental factors, will be vital for developing truly personalized approaches to cerebral malaria. The ultimate goal is to translate these deep mechanistic insights into practical interventions—whether diagnostics, drugs, or supportive therapies—that are safe, effective, and readily deployable in the low-resource settings where cerebral malaria exacts its greatest toll. Achieving this ambitious goal will demand close collaboration among basic scientists, clinicians, engineers, and public health experts, ensuring that advances in understanding directly lead to tangible measures that reduce both mortality and long-term disability from this devastating disease.

## Figures and Tables

**Table 1:** Key biomarkers in cerebral malaria.

Biomarker	Source	Mechanism	Diagnostic/Prognostic Value
Ang-2 ↑/Ang-1 ↓	Endothelium	Tie2 imbalance → BBB leak	Predicts mortality
sICAM-1, sVCAM-1, vWF	Endothelium	Endothelial activation	Correlate with severity
CXCL10 (IP-10)	Endothelium/immune	T-cell recruitment	Predicts fatal CM
PfHRP2	Parasite	Parasite biomass marker	Improves diagnostic accuracy
Extracellular Vesicles	iRBCs, Endothelium	Carry parasite/host signals	Potential precision biomarker

Summary of validated and emerging biomarkers reflecting parasite burden, endothelial dysfunction, and host immune activation. These markers provide diagnostic and prognostic information, and may guide precision medicine approaches in cerebral malaria.

**Table 2:** Translational therapies and targets.

Target Pathway	Candidate Intervention	Evidence
Endothelial stabilization (Ang–Tie2)	Ang-1 mimetics, Tie2 agonists	Preclinical success
Nitric Oxide bioavailability	Inhaled NO	Limited trial benefit
Cytokine modulation	Anti-CXCL10, CXCR3 blockade	Preclinical promise
Inflammasome (IL-33–NLRP3)	IL-33, MCC950	Improves survival in mice
PfEMP1 adhesion	Adhesion blockers	High potential
Cell therapy	MSCs	Neuroprotection in models

Candidate adjunctive interventions targeting vascular stabilization, immune modulation, and parasite adhesion. Preclinical and early translational studies highlight the potential of Angiopoietin–Tie2 modulation, inflammasome inhibition, PfEMP1 adhesion blockade, and cell-based therapies to improve outcomes.

## Data Availability

No new data were created or analyzed in this study.
